# Complete Genome Sequence of a Novel Recombinant Human Norovirus Genogroup II Genotype 4 Strain Associated with an Epidemic during Summer of 2012 in Hong Kong

**DOI:** 10.1128/genomeA.00140-12

**Published:** 2013-02-07

**Authors:** Martin C. W. Chan, Paul K. S. Chan

**Affiliations:** Department of Microbiology, Faculty of Medicine, The Chinese University of Hong Kong, Hong Kong Special Administrative Region, China

## Abstract

Beginning in July 2012, a community-wide increase in the number of norovirus-associated acute gastroenteritis cases was observed during the summer months in Hong Kong. Here, we report the complete genome sequence of a novel recombinant norovirus genogroup II genotype 4 (GII.4) strain, named “2012v,” which is associated with this “early” epidemic outside the usual winter season.

## GENOME ANNOUNCEMENT

Human norovirus is a leading cause of acute nonbacterial gastroenteritis worldwide in all age groups, in both developed and developing countries (1, 2). In the United States, among the food-borne outbreaks that were submitted to the norovirus outbreak surveillance system CaliciNet during 2009–2011, >70% of outbreaks were attributed to norovirus genogroup II genotype 4 (GII.4) strains (3). Although norovirus infections are generally mild and self-limiting in otherwise healthy individuals, severe disease progression may occur, especially in cases caused by GII.4 strains (4). In Hong Kong, two epidemics with exceedingly high norovirus activity outside the winter months were observed in the past decade: one in 2004 (September–October) (5) and another in 2006 (June–July) (6). Notably, the two atypical summer epidemics coincided with the emergence and subsequent predominance of novel GII.4 strains, namely 2004v (Hunter/Sakai) and 2006b (Minerva), named for their respective years.

Beginning in July 2012, a community-wide increase in the number of norovirus-associated acute gastroenteritis cases was observed in Hong Kong (7). In this “early” epidemic, norovirus activity peaked in August and subsided in October—in other words, outside the usual winter season. Here, we report the complete genome sequence of a novel recombinant norovirus GII.4 strain that was associated with this epidemic during the summer of 2012 in Hong Kong.

Viral RNA was extracted from a 10% (wt/vol) stool suspension in phosphate-buffered saline using PureLink viral RNA/DNA mini kit (Life Technologies). The stool specimen was collected at the peak of norovirus activity in mid-August. Purified viral RNA was reverse-transcribed using SuperScript III reverse transcriptase (Life Technologies) with a tagged oligo(dT) primer as we described previously (8). Two overlapping PCR fragments were generated using Phusion High-Fidelity DNA polymerase. The first fragment (~3.4 kb) comprised the complete open reading frame (ORF) 1 except for the RNA-dependent RNA polymerase (RdRp) gene. The second fragment (~4.2 kb) comprised RdRp, viral protein 1 (VP1), and viral protein 2 (VP2) genes, plus a 3′-untranslated region. PCR products were Sanger-sequenced directly using a primer walking approach. The sequence reads were assembled using ChromasPro v.1.7.1 (Technelysium). A detailed PCR protocol and list of primers used are available upon request.

Virus typing and phylogenetic analysis using norovirus typing tool v.1.0 (available at http://www.rivm.nl/mpf/norovirus/typingtool) showed that the epidemic was attributed to a recombinant norovirus strain composed of GII.e RdRp and GII.4 VP1. The VP1 clustered most closely with GII.4 2008 and 2010 strains, with nucleotide and amino acid differences of 5% to 6% and 3% to 4%, respectively; this suggests a new strain (9). The complete genome sequence was designated Hu/GII.4/Hong Kong/CUHK3630/2012/CHN and this novel recombinant GII.4 strain was named “2012v.” An earlier study demonstrated that 10 residues on VP1 (S343, T344, R345, A346, K348, N373, D374, D391, G442, and Y443) of norovirus GII.4 VA387 strain formed the interaction interface with histo-blood group antigens (HBGAs), the putative receptors for human norovirus (10). Notably, the 2012v strain contains a novel N373R mutation on the interaction interface not seen in previous GII.4 strains circulating in Hong Kong and elsewhere before 2012. It is therefore very interesting to know whether the newly emerged 2012v strain targets different susceptible populations.

### Nucleotide sequence accession number.

The norovirus Hu/GII.4/Hong Kong/CUHK3630/2012/CHN sequence was deposited into GenBank under the accession no. KC175323.
